# Genome Sequence of *Acinetobacter lactucae* OTEC-02, Isolated from Hydrocarbon-Contaminated Soil

**DOI:** 10.1128/genomeA.00400-17

**Published:** 2017-05-25

**Authors:** Marco Antonio Rogel-Hernandez, Gabriela Guerrero, Clara Ivette Rincón-Molina, Víctor Manuel Ruiz-Valdiviezo, Crhistian Cisneros-Pérez, José Humberto Castañón-Gonzalez, Aline López-López, Esperanza Martinez-Romero, Reiner Rincón-Rosales

**Affiliations:** a Centro de Ciencias Genómicas, Universidad Nacional Autónoma de México, Cuernavaca, Morelos, Mexico; b Instituto Tecnológico de Tuxtla Gutiérrez, Tecnológico Nacional de México, Tuxtla Gutiérrez, Chiapas, Mexico; c CONACYT Research Fellow-Universidad Autónoma de Tlaxcala, Tlaxcala-Texmelucan, Ixtacuixtla, Tlaxcala, Mexico

## Abstract

*Acinetobacter lactucae* OTEC-02 was isolated from hydrocarbon-contaminated soils. Whole-genome sequence analysis was performed to learn more about the strain’s ability to degrade different types of recalcitrant toxic monoaromatic hydrocarbons. The genome of this bacterium revealed its genomic properties and versatile metabolic features, as well as a complete prophage.

## GENOME ANNOUNCEMENT

The *Acinetobacter* genus belongs to the gamma subclass of *Proteobacteria*. It comprises a diverse group of Gram-negative, strictly aerobic, nonfermenting, nonmotile organisms that are ubiquitous in nature and commonly found in soil (1). The strain *A. lactucae* OTEC-02 was isolated from automotive waste oil-contaminated soil and seems to be nutritionally versatile due to its ability to degrade monoaromatic hydrocarbons like phenol and BTEX (benzene, toluene, ethylbenzene, and xylene).

The OTEC-02 genome was sequenced using the Pacific Biosciences (PacBio RSII) single-molecule real-time (SMRT) sequencing platform. Four SMRT cells of a 15-to-20-kb insert library were sequenced; 168,961 reads, with an average read length of 10,793 bp, were used for the *de novo* genome assembly, which was completed using the program RS_HGAP_Assembly.3 with SMRT Portal Analysis version 2.3.0 (2). One contig of 3,981,712 bp was assembled with a coverage of 392×. Genome annotation was performed using the NCBI Prokaryotic Genome Annotation PipelineGene (http://www.ncbi.nlm.nih.gov/genome/annotation_prok). Clusters of orthologous groups (COGs) were allocated using BLASTx against the COG database, and the hits were accepted with an E value of 1e10 (3). The rRNA operons were verified with RNAmmer (4). The OrthoMCL program was used to obtain the orthologous groups with 30% identity and 60% coverage (5). The PHAST program was used for prophage identification (6). Average nucleotide identity (ANI) was calculated with JSpecies (7).

*A. lactucae* OTEC-02 has a single circular chromosome with an average G+C content of 38.8%. It contains 3,683 putative coding sequences (946 bp average length, 86% coding density), of which 784 have no COG prediction. The comparison between the OTEC-02 and *A. lactucae* NRRLB B-41902 strains showed that both genomes are very similar. The latter was isolated from lettuce in the United States (8). The ANI values between OTEC-02 and NRRL B-41902 were 97.182%, with 89.5% coverage of the genome, confirming that strain OTEC-02 belongs to the species *A. lactucae*. OTEC-02 has six rRNA operons, in contrast to *A. lactucae* NRRL B-41902 which has only one. COG comparison between both strains showed similarities for all categories. Analysis with the OrthoMCL program showed that OTEC-02 has 464 unique genes distributed throughout its genome. The PHAS program identified one 45.8-kb region that represents a complete prophage with 55 open reading frames, 36 of which have matches with the phage protein database. This prophage has proteins similar to 24 different phages, and the best hit was with the lytic *A. baumannii* bacteriophage YMC/09/02/B1251_ABA_BP (NC_019541), with which it shares only 10 proteins (9).

Soils contaminated with hydrocarbons represent an extreme environment. Strains that have the capacity to grow under these conditions have developed different strategies to survive. Analysis of the genome of *A. lactucae* strain OTEC-02 gives us the opportunity to study its capacity for being used in the bioremediation of contaminated soils.

### Accession number(s).

The whole-genome nucleotide sequence of *A. lactucae* OTEC-02 has been deposited in GenBank under the accession number CP020015.
